# Supplementary material for: mTOR contributes to endothelium-dependent vasorelaxation by promoting eNOS expression and preventing eNOS uncoupling
Source: Commun Biol. 2022 Jul 22;5:726. doi: 10.1038/s42003-022-03653-w (PMC9307829; doi:10.1038/s42003-022-03653-w)
Supplement: Supplementary file 2 — Supplementary Information [file 42003_2022_3653_MOESM2_ESM.pdf]

Supplementary Figures

mTOR contributes to endothelium-dependent vasorelaxation by promoting eNOS expression and preventing eNOS uncoupling

Yiying Wang[1,2], Qiannan Li[1,2], Zhiyang Zhang[1,2], Kai Peng[1,2], Dai-Min Zhang[3,4], Qianlu Yang[1,2], Anthony G. Passerini[5], Scott I. Simon[5], ChongXiu Sun[1,2]*

[1]Key Laboratory of Targeted Intervention of Cardiovascular Disease, Collaborative Innovation Center for Cardiovascular Disease Translational Medicine, Nanjing Medical University, Nanjing, China

[2]Key laboratory of Human Functional Genomics of Jiang Province, Nanjing, China

[3]Department of Cardiology, Nanjing First Hospital, Nanjing Medical University, Nanjing, China

[4]Department of Cardiology, Sir Run Run Hospital, Nanjing Medical University, No. 109 Longmian Road, Nanjing 211166, PR China

[5]Department of Biomedical Engineering, University of California Davis, Davis CA, USA

*Correspondence to:
ChongXiu Sun, PhD
Key Laboratory of Targeted Intervention of Cardiovascular Disease, Collaborative Innovation Center for Cardiovascular Disease Translational Medicine, Nanjing Medical University, Nanjing

101 Longmian Avenue, Jiangning District, Nanjing 211166, P.R. China
Tel.: 86-25-86869429
Fax: 86-25-86869429
E-mail: cxsun@njmu.edu.cn

Supplementary Figure 1

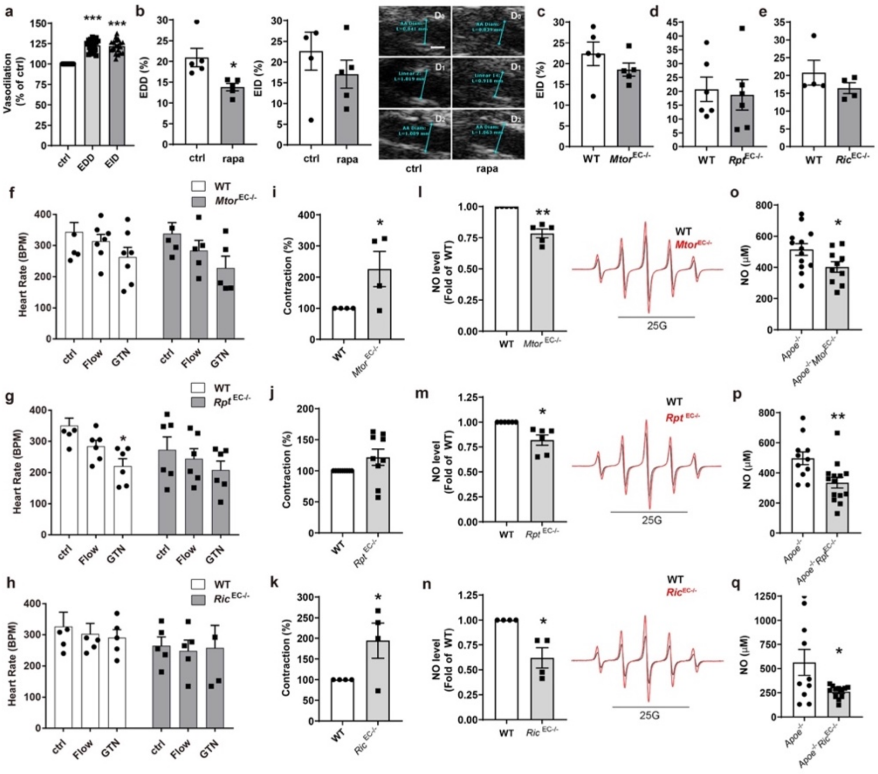

Supplementary Figure 1. Transcutaneous ultrasound was used to measure vascular vasodilatation function. **a,** Flow- and GTN-mediated vasodilatation in WT mice were recorded with transcutaneous ultrasound imaging and EDD and EID quantified (n=21). **b**, Mice were injected i.p. with corn oil (ctrl) or rapamycin (rapa) before endothelium-dependent vasodilation (EDD) and endothelium-independent vasodilation (EID) of the abdominal aortas were measured by transcutaneous ultrasound (n=5) and calculated as follows: EDD%=(D_1-D_0)/D_0×100; EID%=(D_2-D_0)/D_0×100. Where D_0 is the mean diameter of the murine abdominal aorta over three cardiac cycles at baseline, D_1 is the maximum systolic diameter after flow-mediated dilation and D_2 the maximum systolic diameter after nitroglycerin (GTN) infusion. Shown to right are representative images of the abdominal aortas. Scale=0.5 mm. **c-e**, EID of the abdominal aortas of *Mtor*$^{EC-/-}$, *Rptor (Rpt)*$^{EC-/-}$, *Rictor (Ric)*$^{EC-/-}$ mice and their wild type (WT) littermates were measured by transcutaneous ultrasound imaging (n=5-6). **f-h,** Also measured was heart rate which did not display difference between WT and either KO after the same treatment (n=4-6). **i-k,** 10^{-7}mol/L NE induced contraction force in aortic rings of *Mtor*$^{EC-/-}$, *Rap*$^{EC-/-}$, *Ric*$^{EC-/-}$ and their WT littermate mice (n=4-9). **l-n,** NO levels in the serum of *Mtor*$^{EC-/-}$, *Rpt*$^{EC-/-}$ and *Ric*$^{EC-/-}$ mice and their WT littermates (n=4-6) was quantified by the change of cPTIO spectrum measured by an EPR spectrometry. Shown to the right are representative spectra. **o-q**, The serum NO levels in *Apoe*$^{-/-}$*Mtor*$^{EC-/-}$, *Apoe*$^{-/-}$*Rpt*$^{EC-/-}$ *and Apoe*$^{-/-}$*Ric*$^{EC-/-}$ mice and their littermate controls (*Apoe*$^{-/-}$) were measured (n=10-14). Error bars correspond to standard error of the mean (SEM). *p<0.05; **p<0.01; ***p<0.001 vs. ctrl or WT or *Apoe*$^{-/-}$ littermates. Repeated measures (RM) ANOVA with Tukey's posttest (a, f-h) or unpaired two-tailed t test (b-e, o-q) or one sample t test (l-n).

Supplementary Figure 2

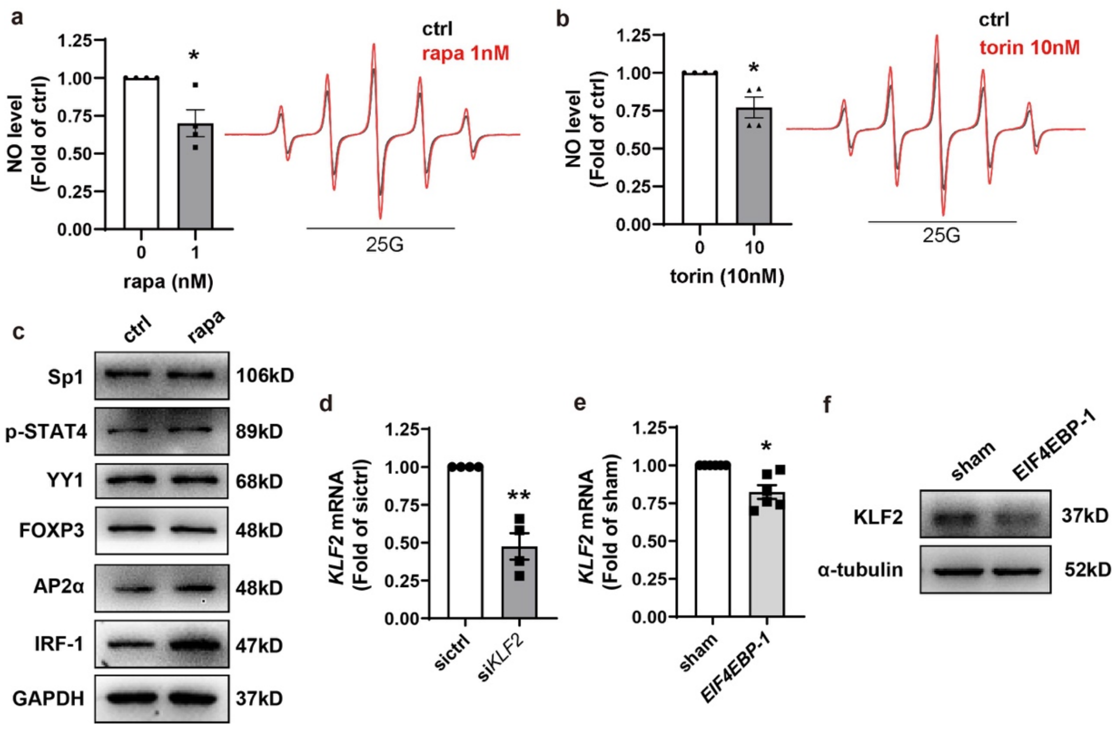

Supplementary Figure 2. Inhibition of mTORC1 decreased KLF2 expression. **a-b,** HAEC were exposed to 1nM rapamycin (rapa, b) or 10nM torin 1 (torin, c) for 1h prior to NO measurement by EPR technique. Shown to the right are presentative EPR spectra images. **c,** HAEC were treated with 1nM rapa prior to Western blot analysis. **d,** HAEC were transfected with *KLF2*-targeting siRNA prior to quantitative PCR (n=4). **e-f,** quantitative PCR (n=6) and Western blot analysis were performed after transfection of pcDNA3.1 *EIF4EBP-1* into HAEC. Error bars correspond to standard error of the mean (SEM). *p<0.05; **p<0.01 vs. ctrl or sictrl or sham; one sample t test.

Supplementary Figure 3

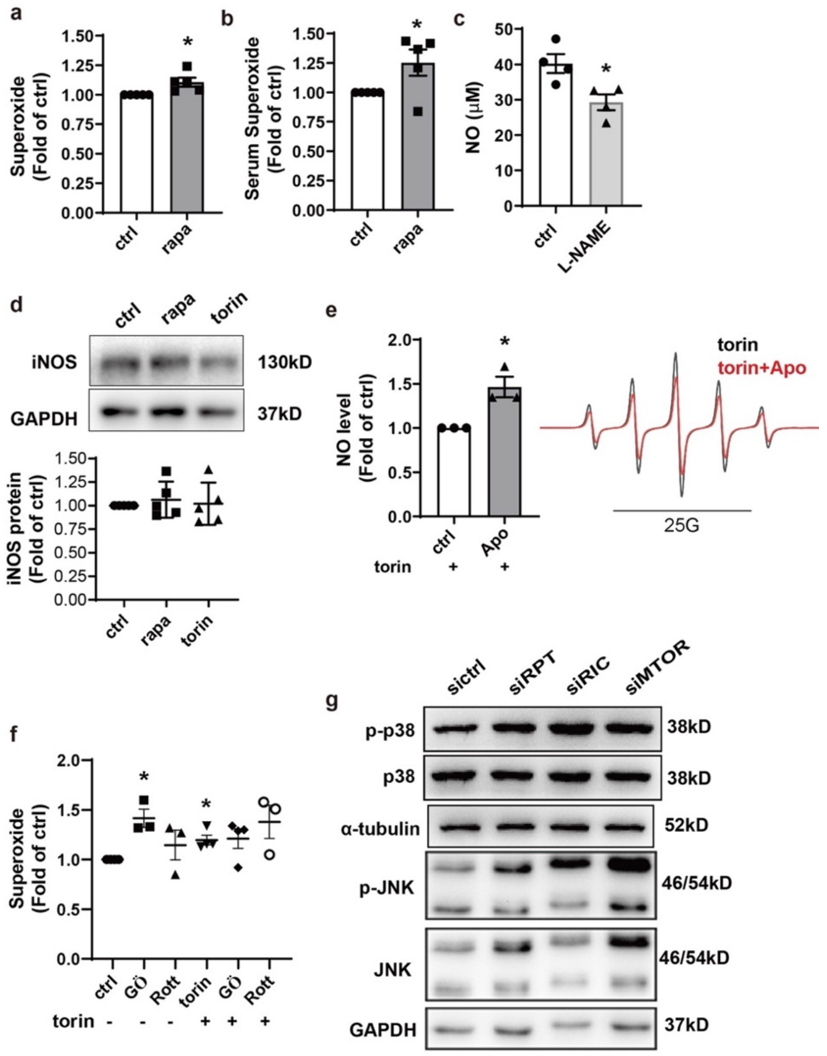

Supplementary Figure 3. Prolonged treatment with rapamycin caused ROS accumulation. **a,** HAEC were treated with 1nM rapa for 48h prior to DHE staining and measurement of ROS with flow cytometry (n=5). **b,** Serum ROS levels were measured in mice administered with vehicle (ctrl) or rapamycin (rapa) with chemiluminescent assay (n=5). **c**, After treated with 500nM L-NAME for 0.5h (n=4), HAEC were submitted to NO assay to confirm the inhibitory effect of L-NAME on NOS. **d**, HAEC were treated with 1nM rapa or 10nM torin for 1h prior to Western blot analysis and quantification of iNOS protein (n=5). **e**, After 1h pretreatment with 20μM Nox inhibitor Apocynin (Apo) (n=3), HAEC were incubated with 10nM torin for 1h prior to ERP measurement of the culture medium. The results confirmed that with chromogenic reaction assay. **f**, HAEC were treated with 5μM Gö-6976 (Gö) or 5μM Rottlerin (Rott) for 1h before incubation with or without 10nM torin for 1h. ROS production was then evaluated with flow cytometry (n=3-4). **g**, The phosphorylation of p38 and JNK1/2 was examined by Western blotting after transfection of siRNA into HAEC. Shown are representative images of four experiments (n=4). Error bars correspond to standard error of the mean (SEM). *p<0.05 vs. ctrl; one-sample t-test, a-b, e; unpaired two-tailed t test, c; RM ANOVA followed by Dunnett's test, d, f.

Supplementary Figure 4

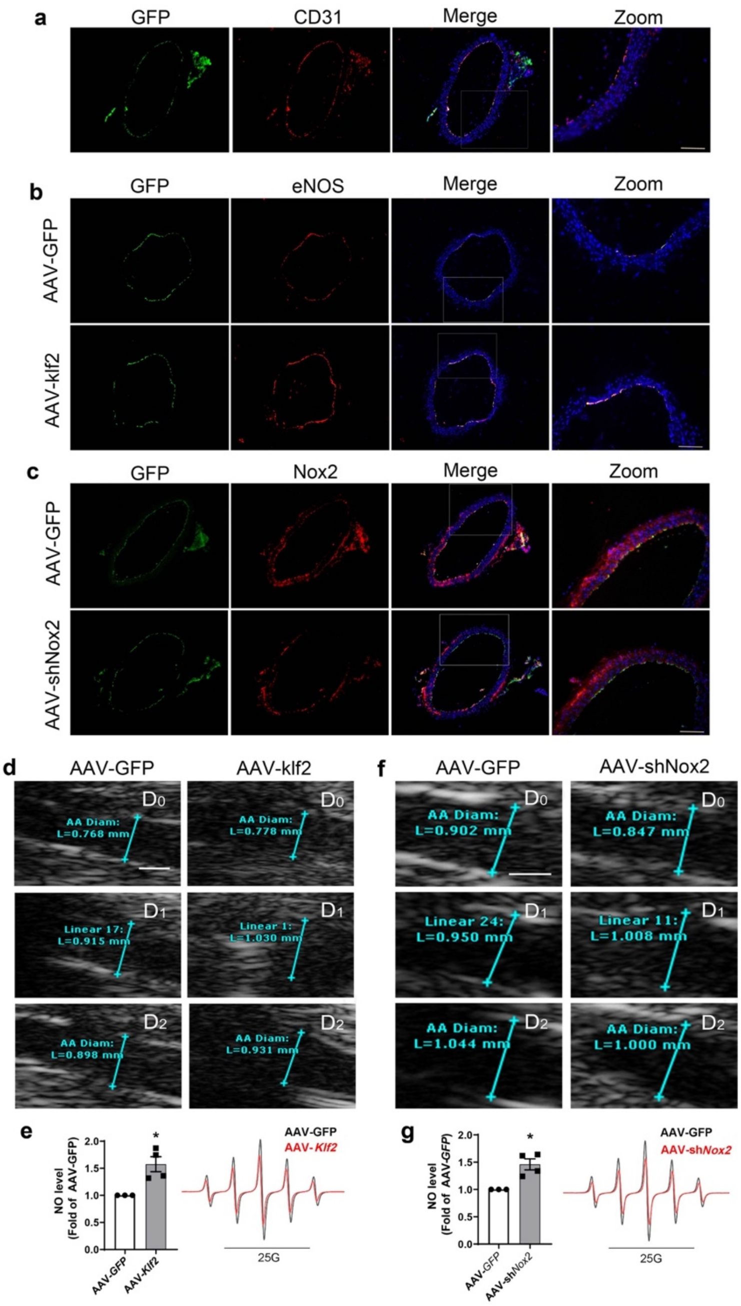

Supplementary Figure 4. *Rptor*$^{EC-/-}$ mice were infected with empty AAV (AAV-EGFP) or AAV-*Klf*2 at a dose of 3×10^{11} vg/mouse while *Rictor*$^{EC-/-}$ mice infected with empty AAV (AAV-EGFP) or AAV-sh*Nox2.* **a,** Fluorescence microscopy analysis of the aorta sections confirmed *ICAM2* promoter-driven EC-specific expression of EGFP as evidenced by its co-localization with CD31. **b,** Infection of *Rptor*$^{EC-/-}$ mice with AAV-*Klf*2 successfully increased the expression of eNOS specifically in the EC. **c**, Nox2 expression in EC was inhibited by infection of AAV-sh*Nox2*. Scale bar, 50 μm. **d,** Representative images of transcutaneous ultrasound assay demonstrated the improved vascular vasodilatation function of *Rptor*$^{EC-/-}$ mice infected with AAV-*Klf*2. Scale=0.5 mm. **e**, *Rptor*$^{EC-/-}$ mice were i.v. injected with AAV-GFP or AAV-*Klf*2 at 3×10^{11}vg. Serum NO was evaluated by EPR assay (n=4). Shown to the right are

representative EPR spectra. **f**, Representative images of transcutaneous ultrasound assay demonstrated the improved vascular vasodilatation function of *Rictor*$^{EC-/-}$ mice infected with AAV-sh*Nox2*. Scale=0.5 mm**. g**, *Rictor*$^{EC-/-}$ mice were injected with 3×10^{11}vg AAV-GFP or AAV-sh*Nox2* followed by evaluation of NO by EPR assay (n=4). *$p<0.05$; one-sample t-test, e, g.

Supplementary Figure 5

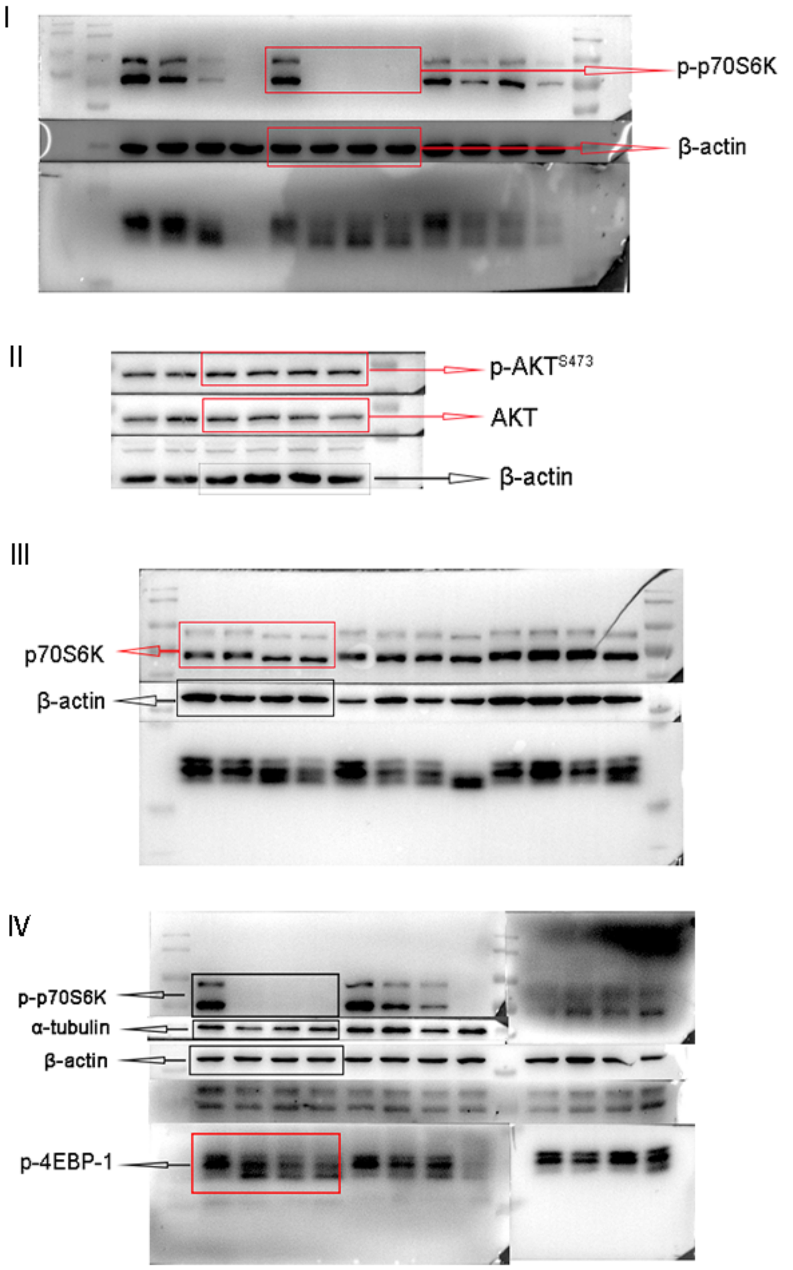

Original Western Blot images for Ⅰ) p-p70S6K, β-actin, Ⅱ) p-AKT^{S473}, AKT, Ⅲ) p70S6K, Ⅳ) p-4EBP-1 in HAEC treated with rapa at indicated dose for 1h. Bands showing in **Figure 2a** are indicated in red rectangles.

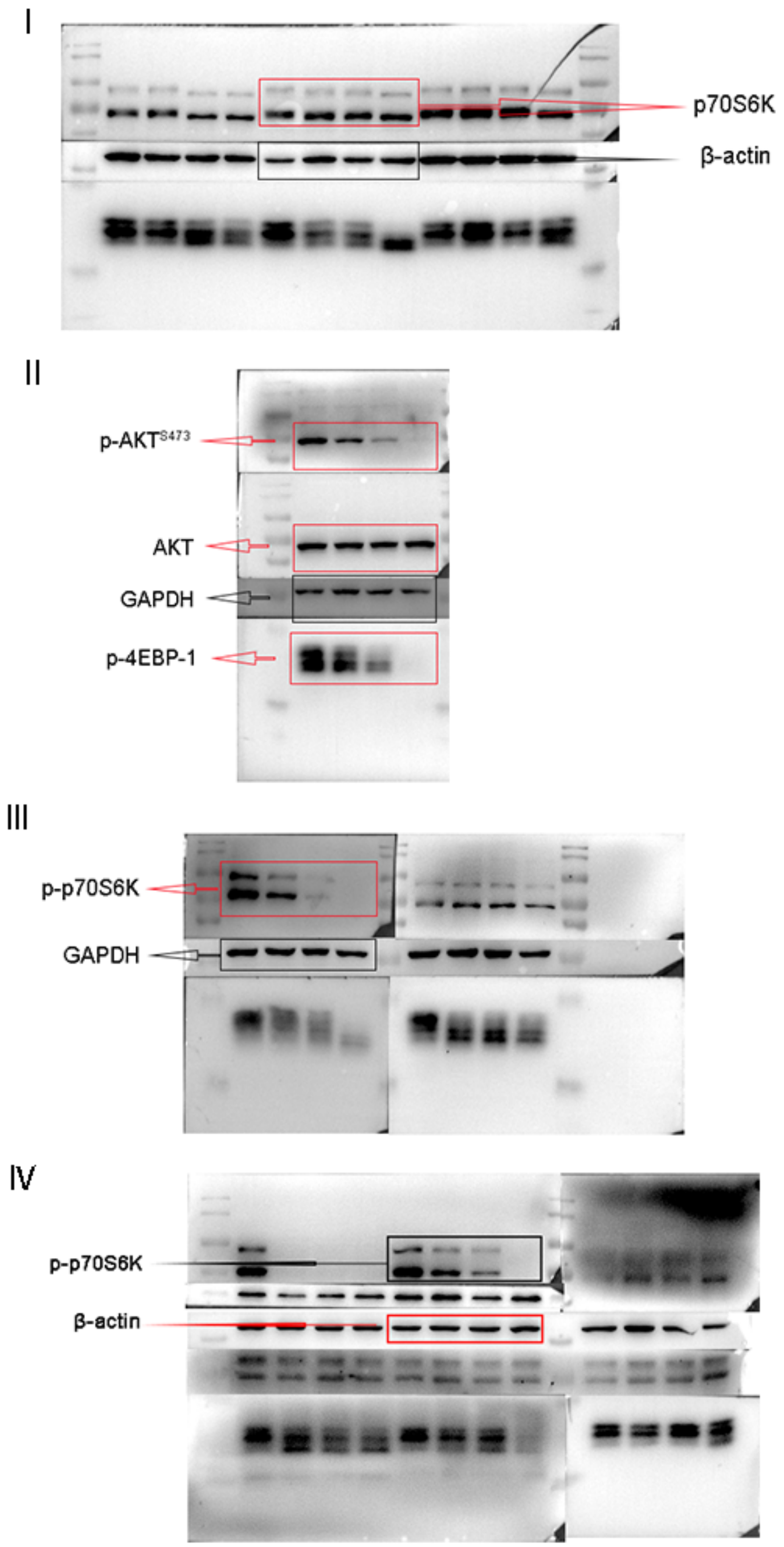

Original Western Blot images for Ⅰ) p70S6K, Ⅱ) p-AKT^{S473}, AKT, p-4EBP-1, Ⅲ) p-p70S6K, Ⅳ) β-actin in HAEC treated with torin at indicated dose for 1h. Bands showing in **Figure 2a** are indicated in red rectangles.

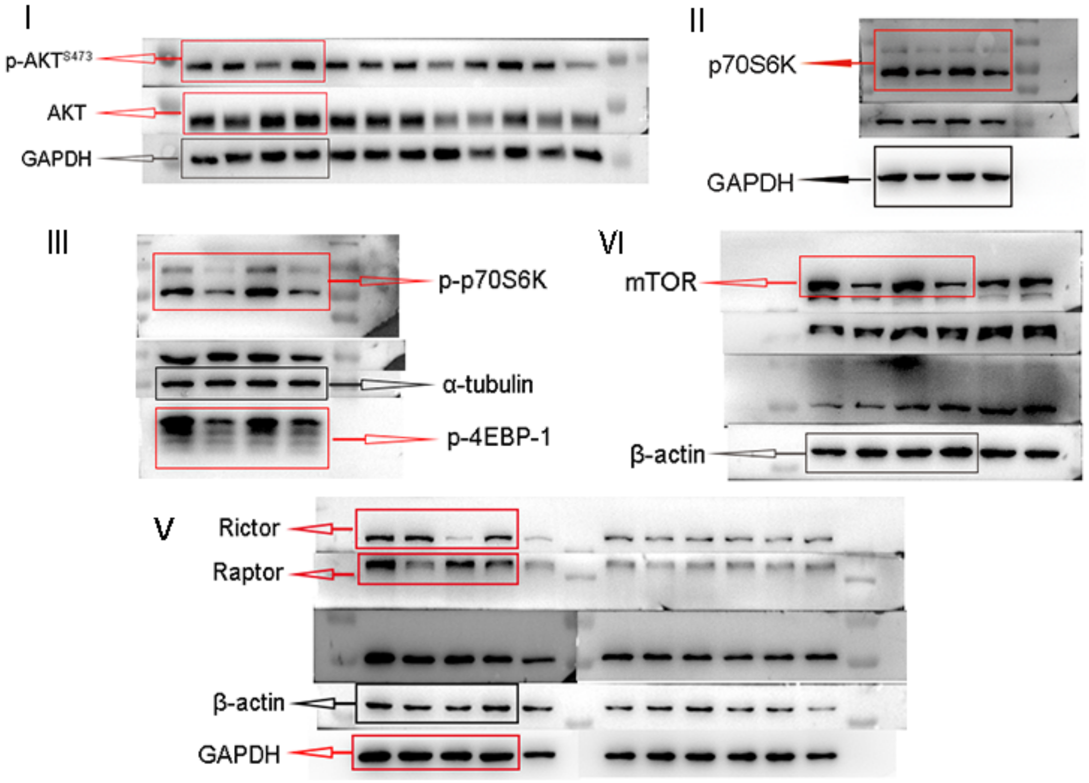

Original Western Blot images for Ⅰ) p-AKT^{S473}, AKT, Ⅱ) p70S6K, Ⅲ) p-p70S6K, p-4EBP-1, Ⅳ) mTOR, Ⅴ) Rictor, Raptor, GAPDH in sictrl-, *siRPT-, siRIC-* or *siMTOR*-transfected HAEC. Bands showing in **Figure 2d** are indicated in red rectangles.

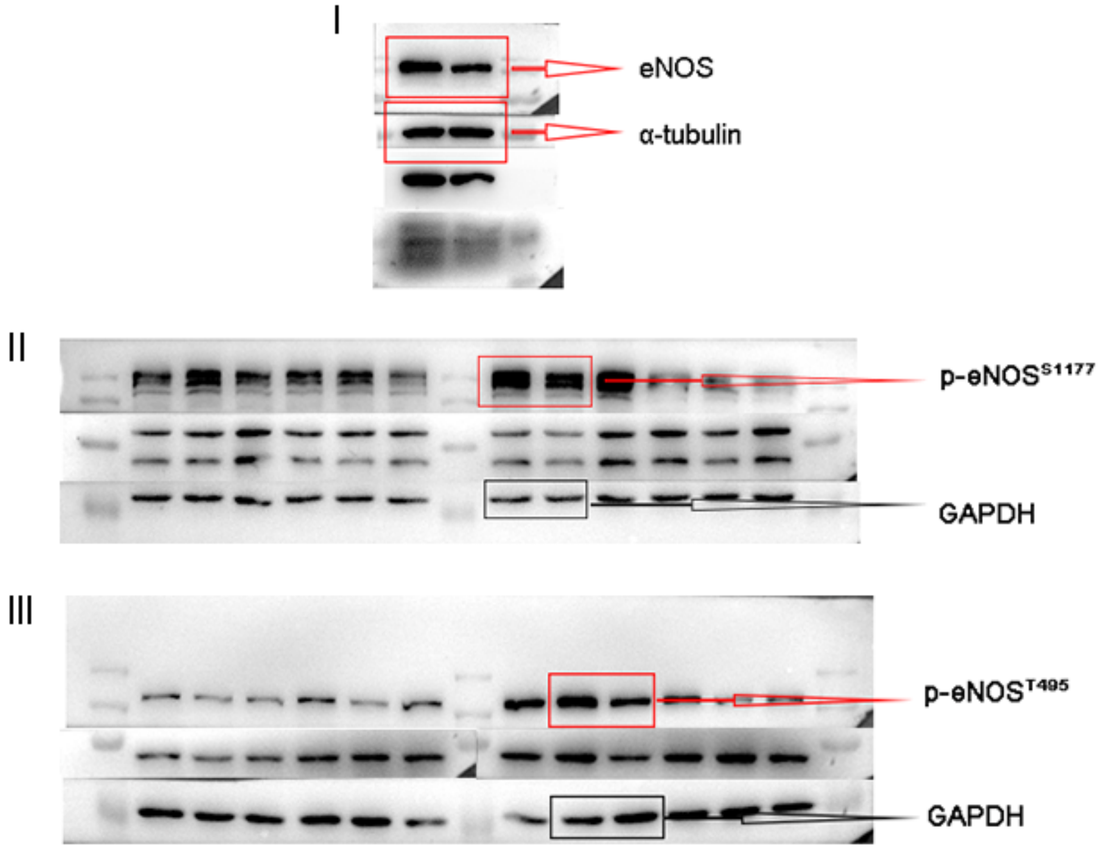

Original Western Blot images for I) eNOS, α-tubulin, II) p-eNOS ^{S1177}, III) p-eNOS^{T495} **in** HAEC treated with 1nM rapa. Bands showing in **Figure 3a** are indicated in red rectangles.

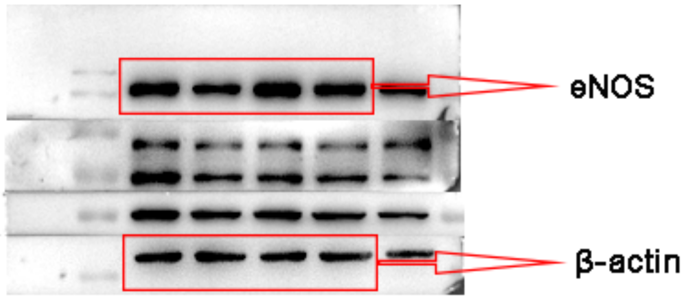

Original Western Blot images for eNOS, β-actin in sictrl, *siRPT, siRIC* or *siMTOR* transfected HAEC. Bands showing in **Figure 3c** are indicated in red rectangles.

I

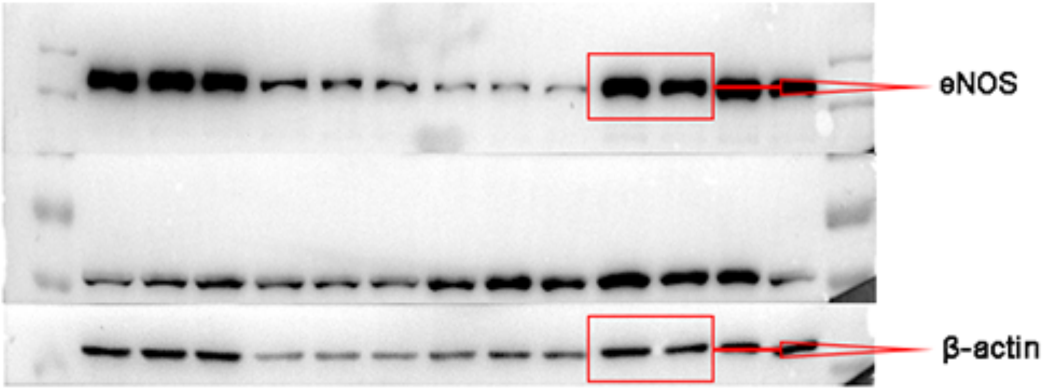

II

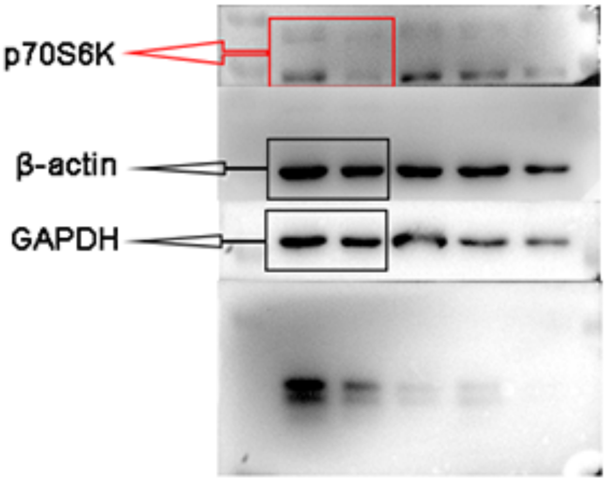

Original Western Blot images for Ⅰ) eNOS, β-actin, Ⅱ) p70S6K in sictrl- or *siRPS6KB1*-transfected HAEC. Bands showing in **Figure 3f** are indicated in red rectangles.

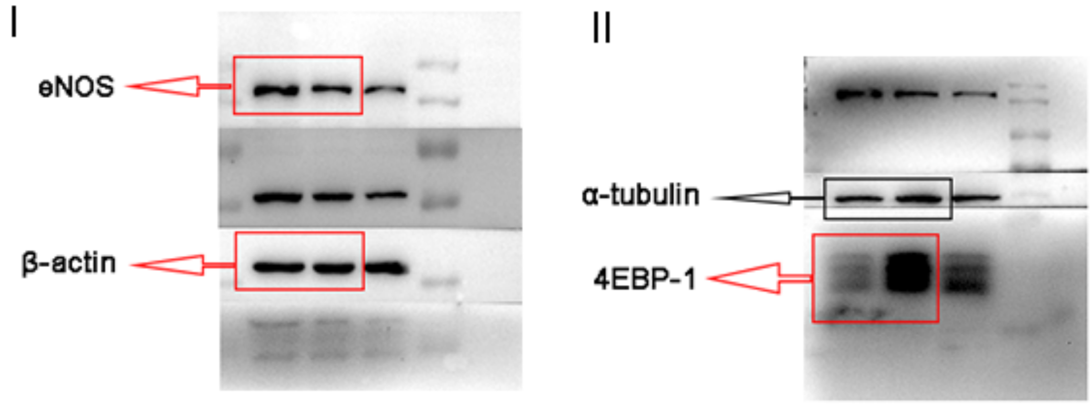

Original Western Blot images for Ⅰ) eNOS, β-actin, Ⅱ) 4EBP-1 in HAEC transfected with pcDNA3.1 (sham) or pcDNA3.1 *EIF4EBP-1* plasmids**.** Bands showing in **Figure 3g** are indicated red rectangles.

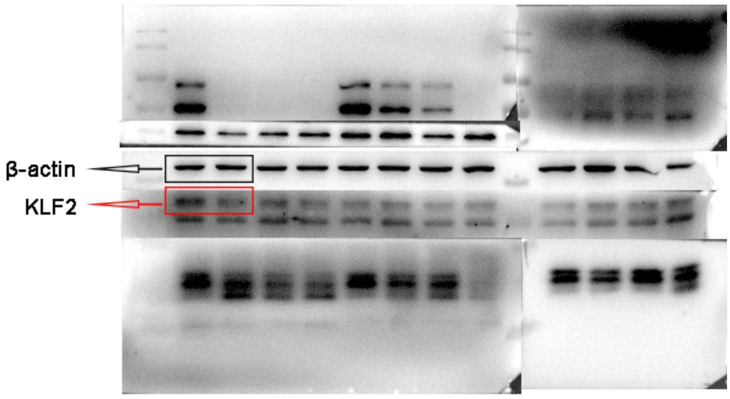

Original Western Blot images for KLF2 in HAEC treated with 1nM rapa. Bands showing in **Figure 4a** are indicated in red rectangles.

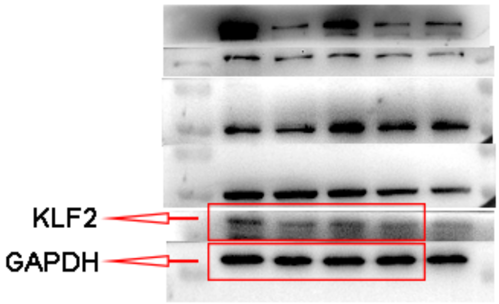

Original Western Blot images for KLF2, GAPDH in sictrl-, *siRPT-, siRIC-* or *siMTOR*-transfected HAEC. Bands showing in **Figure 4b** are indicated in red rectangles.

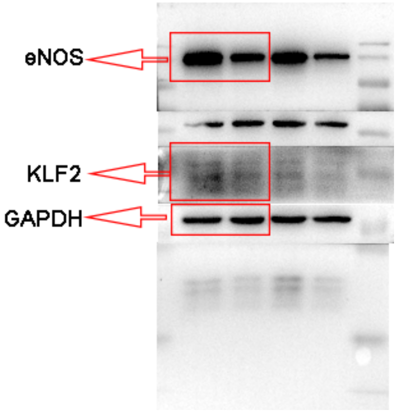

Original Western Blot images for eNOS, KLF2, GAPDH in sictrl or *siKLF2*-transfected HAEC. Bands showing in **Figure 4c** are indicated in red rectangles.

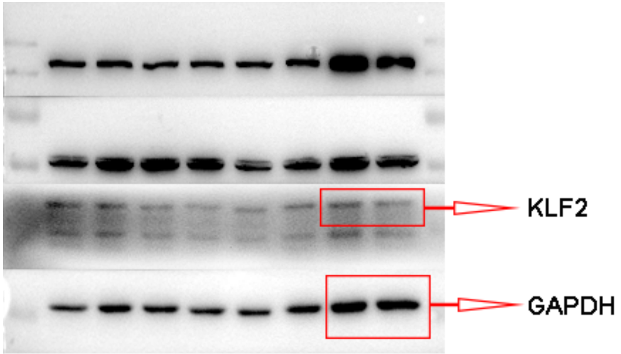

Original Western Blot images for KLF2, GAPDH in sictrl- and *siRPS6KB1*-transfected HAEC. Bands showing in **Figure 4h** are indicated in red rectangles.

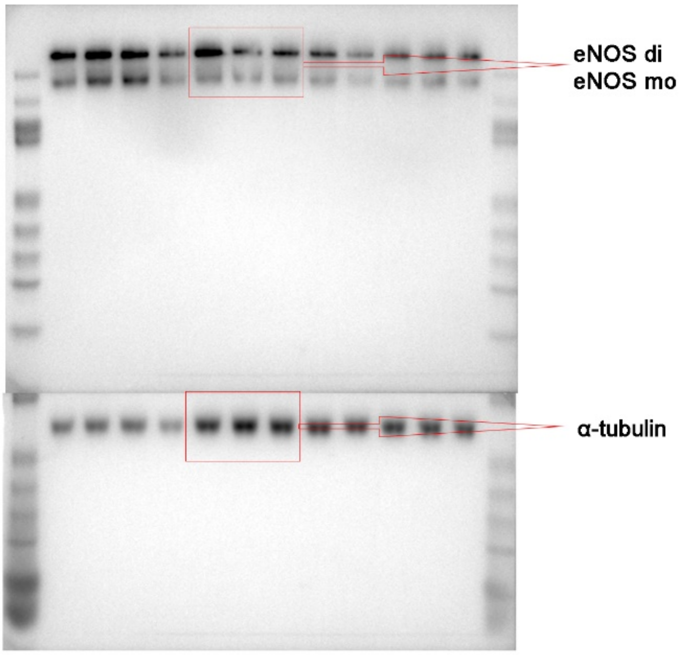

Original Western Blot images for eNOS dimer (di)，monomer (mo)，α-tubulin in HAEC treated with 10nM torin or 1nM rapa for 1h. Bands showing in **Figure 5f** are indicated in red rectangles.

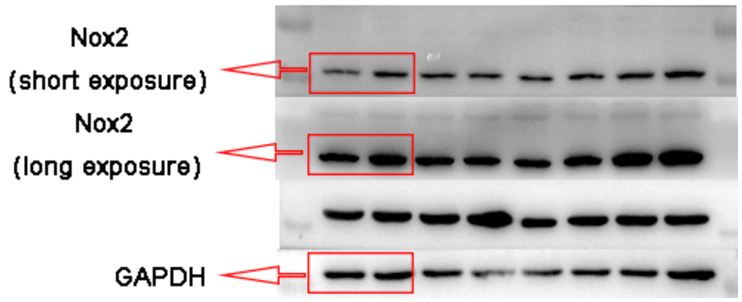

Original Western Blot images for Nox2, GAPDH in HAEC treated with 10nM torin. Bands showing in **Figure 5k** are indicated in red rectangles.

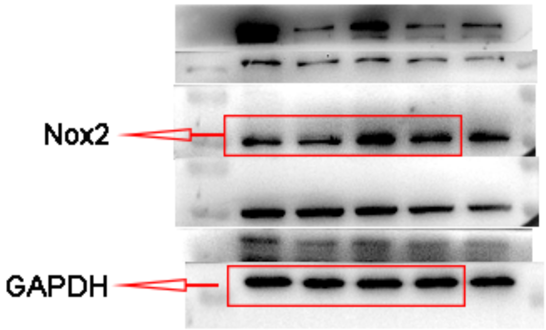

Original Western Blot images for Nox2, GAPDH in sictrl, *siRPT, siRIC* or *siMTOR* transfected HAEC. Bands showing in **Figure 5I** are indicated in red rectangles.

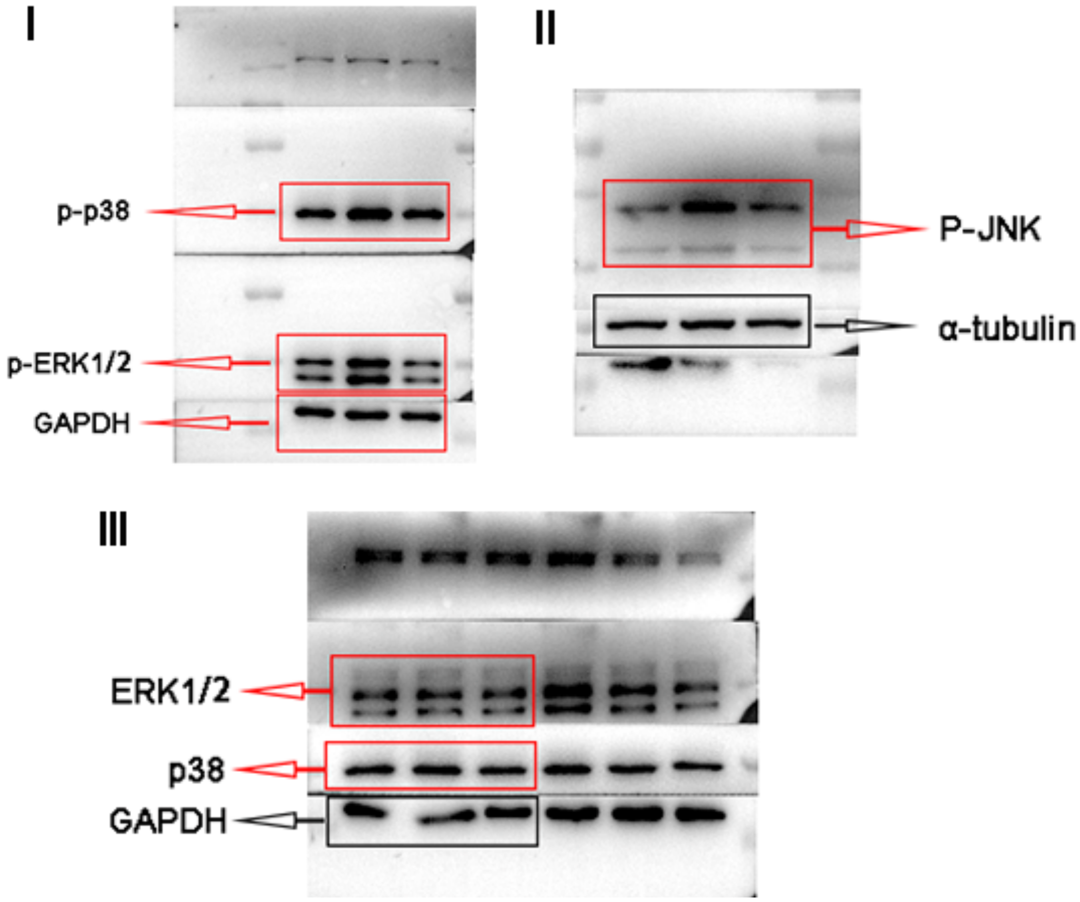

Original Western Blot images for Ⅰ) p-p38, p-ERK1/2, GAPDH, Ⅱ) p-JNK, Ⅲ) ERK1/2, p38 in HAEC treated with 10nM torin or 1nM rapa for 1h. Bands showing in **Figure 6a** are indicated in red rectangles.

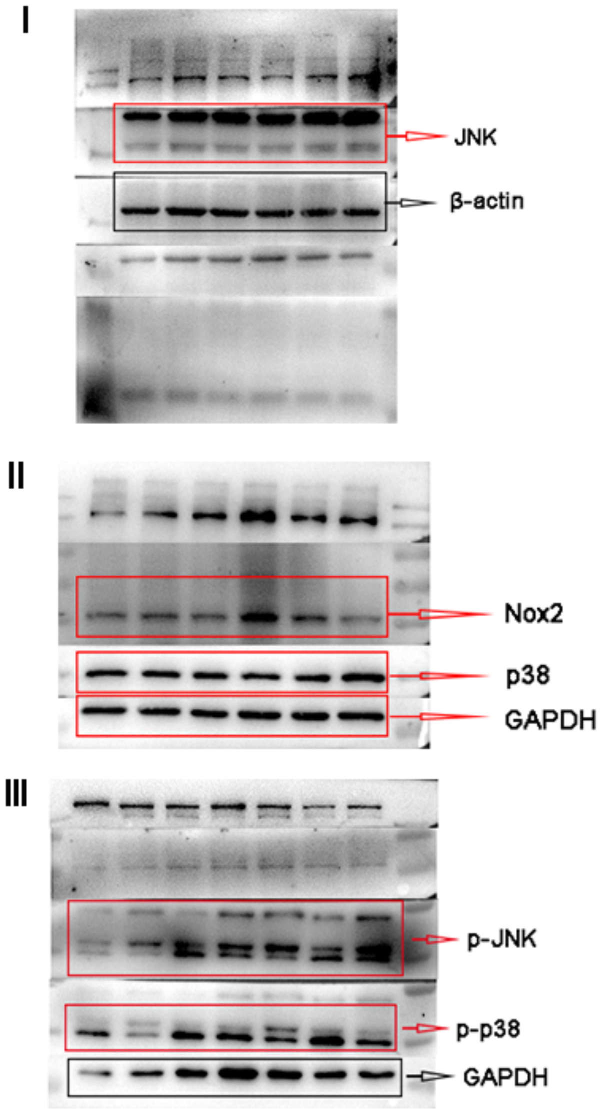

Original Western Blot images for Ⅰ) JNK, Ⅱ) Nox2, p38, GAPDH, Ⅲ) p-JNK, p-p38 in HAEC treated with SB (20μM) or SP (20μM) for 1h before incubation with 10nM torin for 1h. Bands showing in **Figure 6e** are indicated in red rectangles.

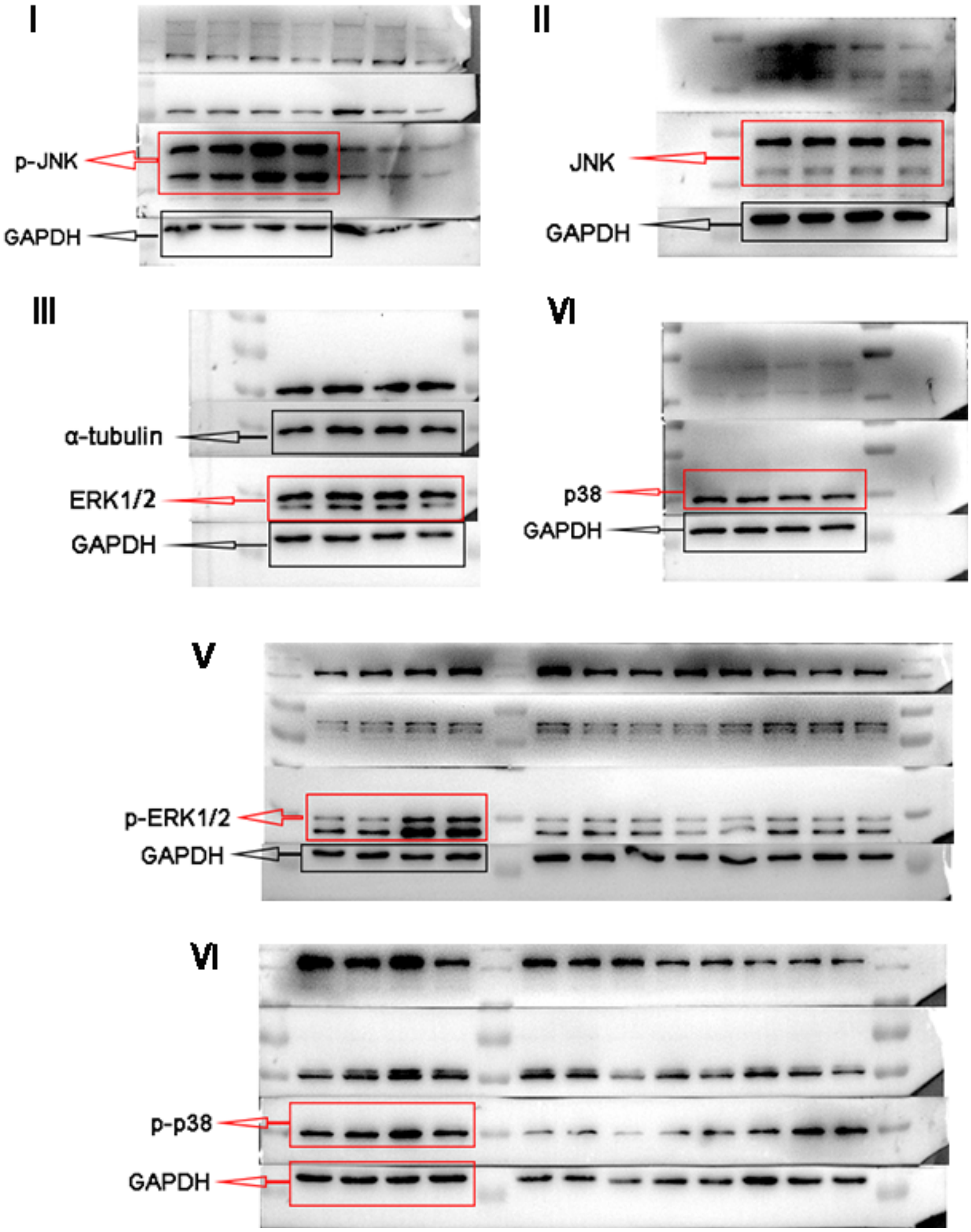

Original Western Blot images for Ⅰ) p-JNK, Ⅱ）JNK, Ⅲ) ERK1/2, Ⅳ) p38, Ⅴ) p-ERK1/2, Ⅵ) p-p38, GAPDH in HAEC treated with APO (20μM) for 1h before incubation with 10nM torin for 1h. Bands showing in **Figure 6f** are indicated in red rectangles.

I

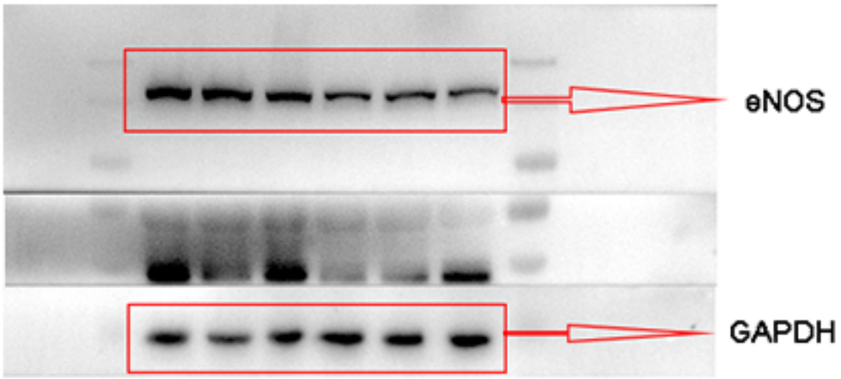

II

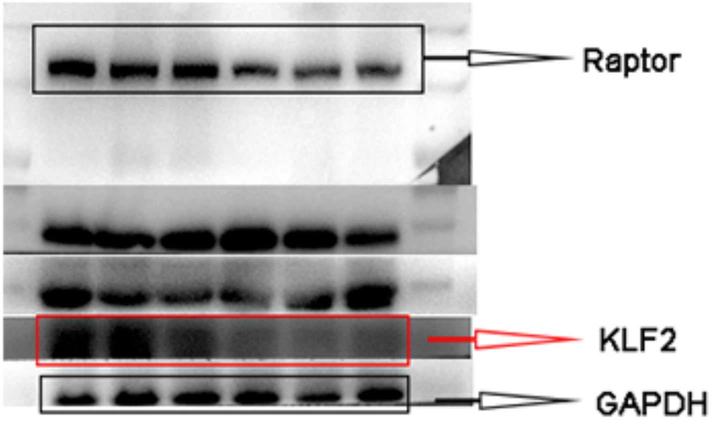

Original Western Blot images for Ⅰ) eNOS, GAPDH, Ⅱ) KLF2 in EC isolated from *Rptor*$^{EC-/-}$ and their WT littermates. Bands showing in **Figure 7a** are indicated in red rectangles.

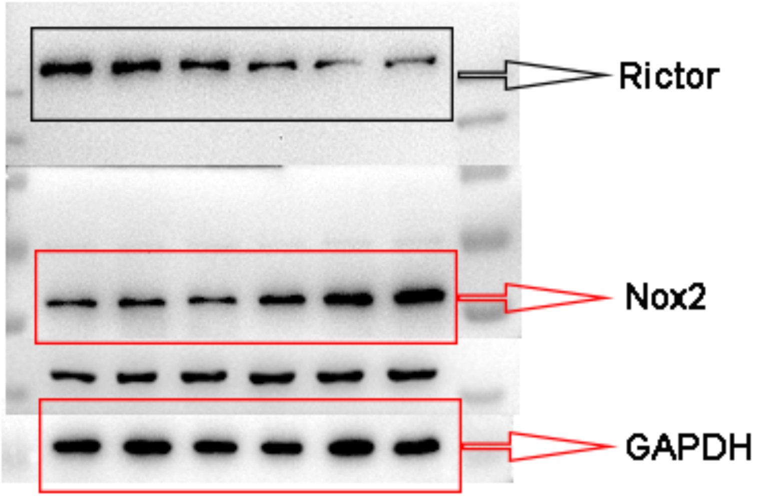

Original Western Blot images for Nox2, GAPDH in EC isolated from *Rictor*$^{EC-/-}$ and their WT littermates**.** Bands showing in **Figure 7b** are indicated in red rectangles.

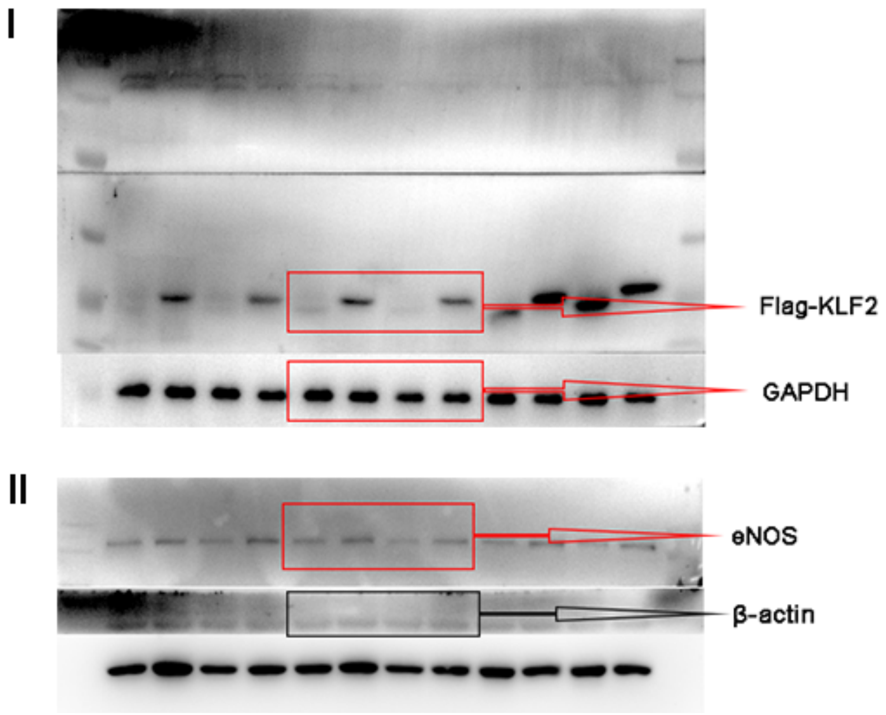

Original Western Blot images for I) Flag-KLF2, GAPDH, II）eNOS in EC isolated from wild type mice were infected with empty AAV (AAV-GFP) or *Klf2*-expressing adeno-associated virus (AAV-*Klf2*) at a multiplicity of infection (MOI) of 10^5vg/cell for 72h, then treated with or without 1nM rapa for 1h. Bands showing in **Figure 7c** are indicated in red rectangles.

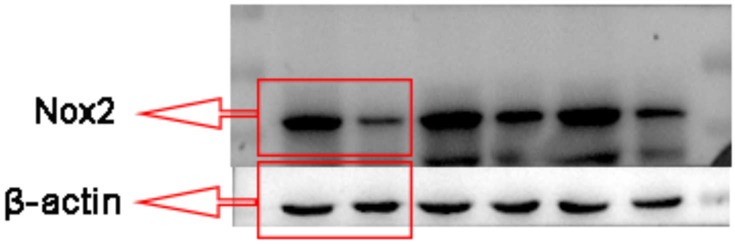

Original Western Blot images for Nox2, β-actin in murine EC infected with AAV containing two tandem mouse Nox2-targeting sequences (AAV-sh*Nox2*). Bands showing in **Figure 7d** are indicated in red rectangles.

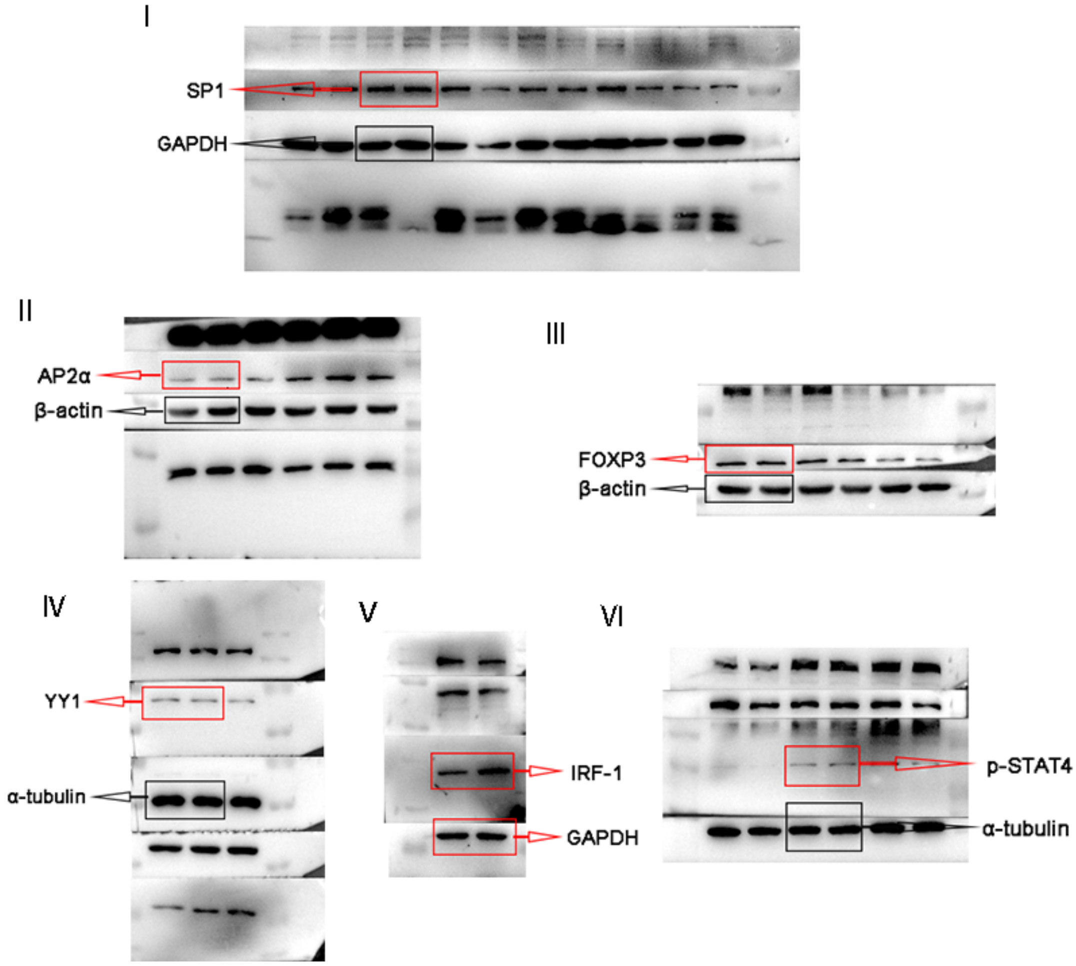

Original Western Blot images for I) SP1, II) AP2α, III) FOXP3, IV) YY1, V) IRF-1, GAPDH and VI) p-STAT4 in HAEC treated with 1nM rapa. Bands showing in **Supplementary Figure 2c** are indicated in red rectangles.

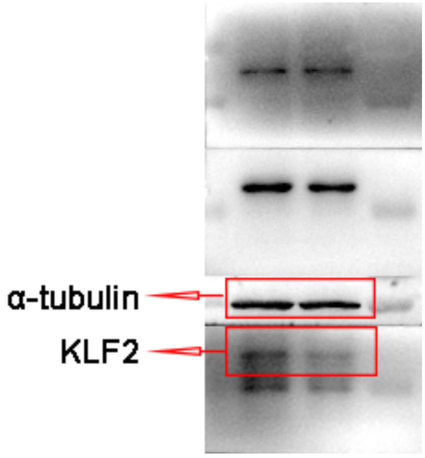

Original Western Blot images for KLF2, α-tubulin in HAEC transfected with pcDNA3.1 (sham) or pcDNA3.1 *EIF4EBP-1* plasmids**.** Bands showing in **Supplementary Figure 2f** are indicated in red rectangles.

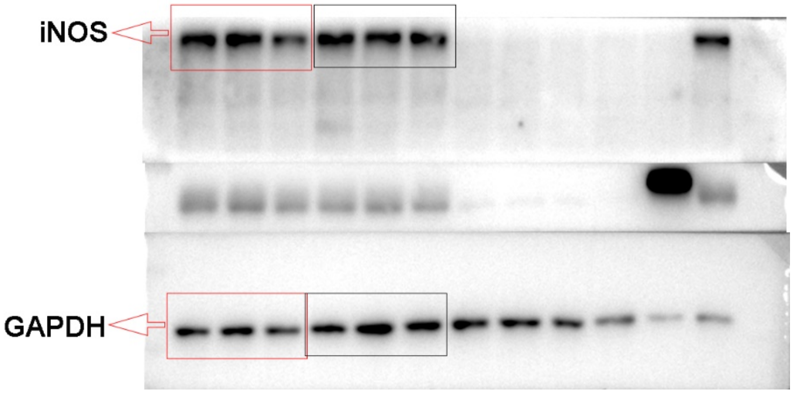

Original Western Blot images for iNOS, GAPDH in HAEC treated with 1nM rapa or 10nM torin for 1h. Bands showing in **Supplementary Figure 3d** are indicated in red rectangles.

I

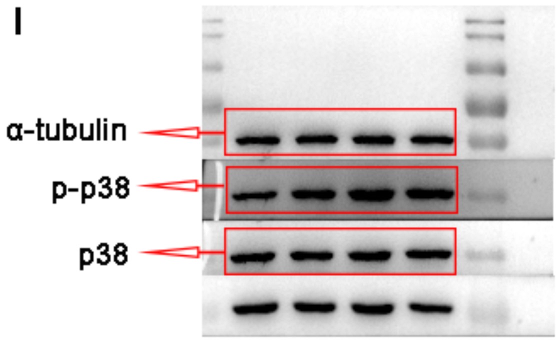

Original Western Blot images for I) p-p38, p38, α-tubulin in sictrl-, *siRPT-, siRIC-* or *siMTOR*-transfected HAEC. Bands showing in **Supplementary Figure 3g** are indicated in red rectangles.
