# Supplementary material for: mTOR contributes to endothelium-dependent vasorelaxation by promoting eNOS expression and preventing eNOS uncoupling
Source: Commun Biol. 2022 Jul 22;5:726. doi: 10.1038/s42003-022-03653-w (PMC9307829; doi:10.1038/s42003-022-03653-w)
Supplement: Supplementary file 3 — Description of Additional Supplementary Files [file 42003_2022_3653_MOESM3_ESM.pdf]

Description of Additional Supplementary Files

File name: Supplementary Data 1
Description: The source data behind the graphs presented in the main figures (in Excel).
