# Supplementary material for: mTOR contributes to endothelium-dependent vasorelaxation by promoting eNOS expression and preventing eNOS uncoupling
Source: Commun Biol. 2022 Jul 22;5:726. doi: 10.1038/s42003-022-03653-w (PMC9307829; doi:10.1038/s42003-022-03653-w)
Supplement: Supplementary file 5 — Reporting Summary [file 42003_2022_3653_MOESM5_ESM.pdf]

nature research

Corresponding author(s): ChongXiu Sun

Last updated by author(s): Feb 8, 2022

Reporting Summary

Nature Research wishes to improve the reproducibility of the work that we publish. This form provides structure for consistency and transparency in reporting. For further information on Nature Research policies, see our Editorial Policies and the Editorial Policy Checklist.

Statistics

For all statistical analyses, confirm that the following items are present in the figure legend, table legend, main text, or Methods section.

n/a	Confirmed	
☐	☒	The exact sample size (n) for each experimental group/condition, given as a discrete number and unit of measurement
☐	☒	A statement on whether measurements were taken from distinct samples or whether the same sample was measured repeatedly
☐	☒	The statistical test(s) used AND whether they are one- or two-sided<br>*Only common tests should be described solely by name; describe more complex techniques in the Methods section.*
☐	☒	A description of all covariates tested
☐	☒	A description of any assumptions or corrections, such as tests of normality and adjustment for multiple comparisons
☐	☒	A full description of the statistical parameters including central tendency (e.g. means) or other basic estimates (e.g. regression coefficient) AND variation (e.g. standard deviation) or associated estimates of uncertainty (e.g. confidence intervals)
☐	☒	For null hypothesis testing, the test statistic (e.g. F, t, r) with confidence intervals, effect sizes, degrees of freedom and P value noted<br>*Give P values as exact values whenever suitable.*
☐	☒	For Bayesian analysis, information on the choice of priors and Markov chain Monte Carlo settings
☒	☐	For hierarchical and complex designs, identification of the appropriate level for tests and full reporting of outcomes
☐	☒	Estimates of effect sizes (e.g. Cohen's d, Pearson's r), indicating how they were calculated

Our web collection on statistics for biologists contains articles on many of the points above.

Software and code

Policy information about availability of computer code

Data collection	GraphPad Prism 7.00 (GraphPad Software, Inc.); FACSCalibur flow cytometer (BD); B-mode ultrasound images (Vevo 770, Visualsonics, Toronto, Canada); Myograph (DMT, Multi Myograph System-620M); digital gel image analysis system (Tanon, Shanghai, China); Light Cycler 480 Instrument II (Roche); SpectraMax Gemini EM microplate spectrofluorometer (Molecular Devices).
Data analysis	GraphPad Prism 7.00 (GraphPad Software, Inc.)

For manuscripts utilizing custom algorithms or software that are central to the research but not yet described in published literature, software must be made available to editors and reviewers. We strongly encourage code deposition in a community repository (e.g. GitHub). See the Nature Research guidelines for submitting code & software for further information.

Data

Policy information about availability of data

All manuscripts must include a data availability statement. This statement should provide the following information, where applicable:

- Accession codes, unique identifiers, or web links for publicly available datasets
- A list of figures that have associated raw data
- A description of any restrictions on data availability

The authors declare that all data supporting the findings of this study are available within the paper and its supplementary information files.

Field-specific reporting

Please select the one below that is the best fit for your research. If you are not sure, read the appropriate sections before making your selection.

☒ Life sciences ☐ Behavioural & social sciences ☐ Ecological, evolutionary & environmental sciences

For a reference copy of the document with all sections, see nature.com/documents/nr-reporting-summary-flat.pdf

Life sciences study design

All studies must disclose on these points even when the disclosure is negative.

Sample size	Both cell samples and animal samples, n>3, satisfy the effective statistical analysis.
Data exclusions	No data was excluded from the analysis.
Replication	All samples were measured in duplicates or triplicates. All the replications appear to be successful.
Randomization	Sample selection complies with the principle of randomness.
Blinding	Investigators were blinded to group allocation during data collection and/or analysis.

Reporting for specific materials, systems and methods

We require information from authors about some types of materials, experimental systems and methods used in many studies. Here, indicate whether each material, system or method listed is relevant to your study. If you are not sure if a list item applies to your research, read the appropriate section before selecting a response.

Materials & experimental systems

n/a	Involved in the study	
☐	☒	Antibodies
☐	☒	Eukaryotic cell lines
☒	☐	Palaeontology and archaeology
☐	☒	Animals and other organisms
☒	☐	Human research participants
☒	☐	Clinical data
☒	☐	Dual use research of concern

Methods

n/a	Involved in the study	
☒	☐	ChIP-seq
☐	☒	Flow cytometry
☒	☐	MRI-based neuroimaging

Antibodies

Antibodies used	Antibodies targeting mTOR (#2983), Rictor (#2114), Raptor (#2280), p-eNOS (S1177) (#9570), IRF-1 (#8478), AP2α (#3215), p-p70S6K (Ser389) (#9234), p70S6K (#9202), p-4EBP-1 (T37/46) (#2855), 4EBP-1 (#9452), p-AKT (S473) (#4060), AKT (#4691), p-p38 (Thr180/Tyr182) (#4511), p38 (#8690), p-ERK (Thr202/Tyr204) (#4370), ERK (#4695), p-SAPK/JNK (Thr183/Tyr185) (#4668), JNK (#9252), GAPDH(#2118), α-Tubulin (#3873) and eNOS (#32027) were from Cell Signaling Technology. Antibodies targeting eNOS (sc-376751), p-eNOS (T495) (sc-136519), gp91phox (sc-130543), Sp1 (sc-17824), p-STAT4 (S721) (sc-28296), YY1 (sc-7341), FOXP3 (sc-166212), Nox2 (sc-130543) were purchased from Santa Cruz Biotechnology. Anti-KLF2 was from Abcam (ab139699) or Bioss (bs-2772r). Antibody targeting iNOS was from Abcam (ab178945). Anti-β-actin was purchased from Bioss (bs-0061r-100). Anti- CD31 was from BD Pharmingen (553370). Alexa 594-conjugated goat anti-rat IgG was from Invitrogen (A11007) and PE-conjugated mouse anti-rabbit IgG from Santa Cruz Biotechnology (sc-3753).
Validation	All antibodies used in this study were commercially purchased from Santa Cruz, CST, Abcam etc and have been validated in this study. Please also refer to the manufacturer's website for validation statement.

Eukaryotic cell lines

Policy information about cell lines

Cell line source(s)	Primary HAEC were purchased from American Type Culture Collection (Cat No. PCS-100-011, Lot No. 63233442Manassas, VA).
Authentication	Authenticated by American Type Culture Collection.
Mycoplasma contamination	None.

Commonly misidentified lines
(See ICLAC register)

N/A

Animals and other organisms

Policy information about studies involving animals; ARRIVE guidelines recommended for reporting animal research

Laboratory animals	Apoe-/- (002052), Mtorflox/flox (011009), Rptorflox/flox and Rictorflox/flox mice on a C57BL/6 genomic background were from the Jackson Laboratory. EC Cdh5 promoter-driven CreERT2 mouse line (Cdh5CreERT2) on C57BL/6 background was developed by Dr. Ralf Adams1 and generously provided by Dr. Bin Zhou at Shanghai Institute of Biochemistry and Cell Biology, Chinese Academy of Sciences. Mtorflox/flox?Rptorflox/flox and Rictorflox/flox mice were crossed with Cdh5CreERT2 mice. Both EC-specific knockout mice (positive for Cdh5CreERT2 allele, also referred as Mtor EC-/-?Rptor EC-/- and Rictor EC-/-) and their wild type littermate controls (negative for Cdh5CreERT2 allele, referred as WT) at 6-week old received intraperitoneal injections of tamoxifen (135mg/kg, dissolved in corn oil) for 7 consecutive days to induce EC-selective deletion of Mtor, Rptor or Rictor in Mtor EC-/-?Rptor EC-/- or Rictor EC-/- mice.
Wild animals	N/A
Field-collected samples	N/A
Ethics oversight	All animal procedures were approved by the institutional animal care and use committee (IACUC-1908030).

Note that full information on the approval of the study protocol must also be provided in the manuscript.

Flow Cytometry

Plots

Confirm that:

- [ ] The axis labels state the marker and fluorochrome used (e.g. CD4-FITC).
- [ ] The axis scales are clearly visible. Include numbers along axes only for bottom left plot of group (a 'group' is an analysis of identical markers).
- [ ] All plots are contour plots with outliers or pseudocolor plots.
- [x] A numerical value for number of cells or percentage (with statistics) is provided.

Methodology

Sample preparation	The intracellular O2-. was evaluated using a fluorescent dye dihydroethidium (DHE, S0063, Beyotime Institute of Biotechnology) as previously described.6 After treatment, HAEC were harvested, washed twice with PBS, and incubated with 2.5μM DHE for 30min at 37°C in dark. The intracellular accumulation of O2-. was then detected with FACSCalibur flow cytometer (BD) followed by analysis with FlowJo.
Instrument	FACSCalibur flow cytometer (BD)
Software	FlowJo software
Cell population abundance	Almost 100% of cell population are positive for intracellular O2- although at varied degree.
Gating strategy	All cells excluding cell debris.

- [x] Tick this box to confirm that a figure exemplifying the gating strategy is provided in the Supplementary Information.
